# The Italian Face-Name Association Test (ItFNAT): a preliminary validation of three parallel versions

**DOI:** 10.1007/s10072-025-08165-z

**Published:** 2025-04-21

**Authors:** Valerio Manippa, Alessandro Oronzo Caffò, Davide Rivolta

**Affiliations:** Department of Education, Psychology and Communication, University of Bari Aldo Moro, Bari, Italy

**Keywords:** Neuropsychology, Cognitive assessment, Neurodegenerative disease, Face-Name associative memory exams

## Abstract

**Background and objectives:**

Associating names with faces is crucial for social interactions and reflects cognitive health. To address the need for reliable tools to assess associative memory, we developed and validated the Italian Face-Name Associative Test (ItFNAT), a tool allows clinicians to monitor cognitive functioning and detect early signs of cognitive decline.

**Materials and methods:**

101 Italian participants (51 females) aged 18–80 years completed the three parallel versions of the ItFNAT, which assessed immediate recall (IR), delayed free recall (DFR), and delayed recall with cues (DTR). ItFNAT was administered alongside other neuropsychological tests to explore its relationship with memory and attention.

**Results:**

ItFNAT demonstrated high internal consistency across its three versions. Principal Component Analysis revealed that IR, DFR, and DTR loaded strongly onto a single factor in each version. Kruskal-Wallis ANOVA indicated no significant differences in scores across versions. Non-parametric analyses showed that years of education significantly influenced all three scores, while age negatively correlated with DTR. Spearman’s correlations revealed strong associations between ItFNAT scores and other widespread memory and attentive tests.

**Discussions:**

This study introduces the ItFNAT, a test designed to assess cross-modal associative memory. It includes three parallel versions with good internal consistency, and minimal score differences. Scores—IR, DFR, and DTR—reflect a shared underlying cognitive construct, correlating with both traditional memory tests and scales assessing working memory and attention. Education significantly influenced all three scores, while age negatively impacted DTR. Future research should refine its application for tracking cognitive function and detecting neurodegenerative changes.

## Introduction

The ability to associate a name with a specific face is a critical aspect of social cognition, and it is essential for recognizing and interacting with others in daily life. Difficulties in learning new names or associating the correct name with a familiar face represent a rather common cognitive complaint among older adults [17, 36]. This process requires the integration of complex ecological episodic memory, involving both visual and verbal processing, making it a strong indicator of overall cognitive function. Albeit various tests have been developed to assess cross-modal associative memory for specific experiments (e.g., Sperling et al., [32, 34], the first standardized version of a face-name memory test was introduced by Rentz et al. [26]. In this version, participants are required to remember 16 unfamiliar face-name pairs and 16 face-occupation pairs. The test consists of an initial study phase, followed by an immediate free recall trial and a recognition trial. Today, such tests are commonly referred to as Face-Name Associative Memory Exams (FNAME). Although alternative versions have been developed and standardized since Rentz’s original test, they all adhere to a similar structure typically comprises three phases: face-name encoding (study), an immediate free recall trial, and a delayed recall trial (free recall and recognition).

Despite differences in administration procedures, numerous studies have demonstrated the efficacy of the FNAME in helping the diagnosis of neurodegenerative diseases such as Alzheimer’s Disease (AD) [27, 37], underscoring its importance for early detection. Recently, an alternative version of the FNAME called Face-Name Association Test (FNAT) has been used to evaluate the impact of a session of transcranial alternating current stimulation (tACS) on cognitive functioning in AD and Mild Cognitive Impairment (MCI) due to AD [3, 4]. Their study demonstrated the test’s reliability in detecting cognitive improvements resulting from tACS. Unlike the original FNAME developed by Rentz (2011), Benussi et al. [3] employed a computer-administered version featuring 20 face-name associations (excluding face-occupation associations) studying, followed by a retrieval phase with faces either presented without cues or, in case of missed/wrong response, with cues. Other versions of the FNAME/FNAT have been used in Italian samples to test various experimental conditions [2, 12], but none of these versions were standardized.

Given the lack of validated versions in Italy, the use of instruments investigating face-name associative memory is currently limited to experimental settings, despite its potential to provide valuable insights into degenerative processes related to associative memory [27]. Furthermore, it remains unclear how this tool assesses cross-modal associative memory or general cognitive functioning; this gap has significant implications for both clinical practice and research. To address this, we have developed and validated three parallel versions of the Italian FNAT (ItFNAT). Each version was administered as part of a neuropsychological battery to evaluate whether ItFNAT scores correlate with commonly used tests that assess memory and attentional processes. Our goal is to standardize ItFNAT procedure and establish preliminary normative data, ensuring its reliability and validity within the Italian context. Additionally, by providing three parallel versions of the ItFNAT, we aim to offer clinicians an easy-to-use tool that can track both normal and pathological aging while minimizing potential learning effects.

## Materials and methods

### Participants

One-hundred-one Italian participants (51 F) were included in this study (Age range: 18–80 years, *M*_age_: 32.12 years, *SD*_age_: 15.34 years; Years of Education range: 5–21 years, *M*_education_: 14.59, *SD*_education_: 3.30 years) via snowball sampling. In the initial recruitment phase, participants were recruited and randomly assigned to the three versions of the ItFNAT. Subsequently, a targeted selection was applied to ensure a balanced distribution of age, education, and gender across the three parallel versions (see Table 1). All participants provided informed consent before completing the procedure. Exclusion criteria were knowing the administered tests, history of neurological diseases, cerebral stroke, epilepsy or epileptic seizures, head injury with loss of consciousness, severe medical conditions or psychiatric disorders, and alcohol or drug abuse. All participants had normal or corrected-to-normal vision. The study was approved by the Ethical Committee of the Institution and was performed following the Helsinki Declaration and its later amendments.

**Table 1:** Descriptive statistics and p-values for age, education, and sex distribution across the three versions of the ItFNAT (v1, v2, and v3). The p-values are from one-way ANOVAs for age and education, and a chi-square test for sex distribution, indicating no significant differences

	ItFNAT v1 (*N* = 34)	ItFNAT v2 (*N* = 33)	ItFNAT v3 (*N* = 34)	
	*M ± SD*	*M ± SD*	*M ± SD*	*p-value*
Age (in years)	32.79 ± 17.52	32.33 ± 15.40	32.33 ± 13.23	0.913
Education (in years)	14.06 ± 3.73	14.18 ± 2.72	15.53 ± 3.29	0.128
Sex (N)	F = 20, M = 16	F = 16, M = 17	F = 15, M = 19	0.461

### ItFNAT

In each parallel version of the Italian Face-Name Association Test (ItFNAT), we associated 16 black-and-white faces with 16 names. Half of the pairs were male, and the other half were female. Faces were extracted from the neutral frontal-view faces of the Karolinska Directed Emotional Faces database [15], while names (both correct and distractor used for the recognition) were chosen among the most common name spread within Italian population during 2022 (Istat, 2023). ItFNAT was administered using Microsoft Office PowerPoint, and the responses were collected by post-graduate psychologists using ad-hoc scoring grids. All the materials and full instructions are free available at: 10.6084/m9.figshare.28113620.

The administration including encoding, immediate recall, and delayed recognition, emulate those used in the most widely shared and common long-term memory tests. Particularly, the test was divided into three phases:

1. During the *encoding* phase, participants were asked to view and memorize 16 faces, each presented one at a time for 2 s. Following this, each face, presented in a different order than the previous presentation, was paired with a name. Participants were asked to read the name aloud to memorize and associate it with the face shown. Participants then moved on to the next face-name pair at their own pace.
2. During the *immediate recall* phase, participants were shown all faces, one at the time, and asked to recall the name paired with it during the encoding phase (immediate recall). After participant’s response (within a maximum 10 s), the correct name was shown again, and they were asked to try to memorize it if they had initially missed or failed to recall it. Then, the next face was presented.
3. After 15 min, during which participants completed other tests that did not require the memorization of verbal information, the *delayed recall* phase began. In this phase, a face was presented on the left side of the screen, and participants were asked to recall the associated name (delayed free recall) within a maximum 10 s. If they miss or fail to recall the name, three choices were shown on the right of the faces (recognition): the correct name, a name previously associated with another face (interference), or a name that had not been presented before (intrusion). After the recognition response, the next face was presented.

The first and second phases are consecutive and last about 10 min. After 15 min of interval, the third phase (delay recall) lasts about 5 min.

A score of 1 was assigned for each correct answer, while missed or incorrect responses received a score of 0. Main scores were:

1. Correct Immediate Recall (IR): the number of name-face pairs correctly recalled during the immediate recall trial;
2. Correct Delayed Free Recall (DFR): the number of name-face pairs correctly recalled during the delayed free recall trial;
3. Delayed Total Recall (DTR): the sum of name-face pairs correctly recalled during the delayed recall trial (free and recognition).

### Procedure

Participants were administered a neuropsychological battery consisting of the ItFNAT and the Italian version of the following tests:

- Digit Span (Forward and Backward; Monaco et al., [22]: This test assesses short-term and working memory. In the Forward task, participants repeat a sequence of numbers in the same order they were presented. In the Backward task, participants repeat the numbers in the reverse order. The number of digits in each sequence (span length) increases by one digit every two trials. In our study we used the numbers of sequence correctly repeated by the participant, with separate scores for Forward (DS-F) and Backward (DS-B) tasks.
- Rey Auditory-Verbal Learning Test (RAVLT; Caltagirone et al., [9]: This test evaluates episodic memory and learning by having participants listen to a list of words, recall them immediately (immediate recall), and after a delay (delayed recall), assessing both retention and retrieval abilities. The score is the number of words correctly recalled in immediate and delayed recall trials, with additional scores for recognition.
- Trail Making Test (TMT; Giovagnoli et al., [14]: This test measures visual attention and task-switching. It consists of two parts: TMT-A, where participants connect numbered circles in order, and TMT-B, where they alternate between numbers and letters. The score we used in our analyses is the time taken to complete TMT-A and TMT-B, separately.
- Rey-Osterrieth Complex Figure Test (ROCF; Caffarra et al., [7]: This test assesses visuospatial abilities and memory. Participants are asked to copy a complex geometric figure and then reproduce it from memory after a delay. The score is based on the accuracy and completeness of the copied (copy) and recalled (recall) figures, typically using a standardized scoring system.
- Everyday Memory Questionnaire (EMQ; Calabria et al., [8]: This self-report questionnaire evaluates the frequency of memory failures in daily life, providing insights into the practical implications of memory performance. The score is the sum of responses to the questionnaire items, with higher scores indicating more frequent self-reported memory lapses.
- Stroop Color and Word Test (SCWT; [30]): This test, which features various subtests and comes in different versions, assesses attention, cognitive control, and executive function. Specifically, participants are asked to name the color of the ink in which color words are written. This latter can either match or mismatch the color word (e.g., the word “Blue” written in green ink, representing an incongruent condition) requiring the inhibition of the automatic reading response. For our analysis, we used the time taken and the number of errors in the incongruent condition as measures of cognitive control.

The order of the tests varied among participants. However, the sequence was designed so that there was about 15-minute interval between the immediate recall and the delayed recall of the RAVLT and the ItFNAT. During this interval, participants completed tests that did not require memorizing materials (i.e., TMT, ROCF copy, EMQ or SCWT).

## Data analysis and results

First, three Principal Component Analyses (PCAs) were conducted to explore the underlying structure of each ItFNAT version. PCAs were based on the three scores of each version. It was not possible to perform PCAs based on the items of each score, due to limited sample size [18, 20]. Results are summarized in Table 2 and revealed a unidimensional structure for each version with a single component having an eigenvalue ranging from 2.167 (Version 2) to 2.411 (Version 1) and explaining a proportion of the total variance varying from 0.722 (Version 2) to 0.804 (Version 1). The three ItFNAT scores (IR, DFR, DTR) loaded strongly onto a single principal component in each version, with loadings ranging from 0.788 (DTR, Version 3) to 0.955 (DFR, Version 1), indicating that the scores of each version share a substantial amount of variance among them. The uniqueness values ranged from 0.088 (DFR, Version 1) to 0.380 (DTR, Version 3). Then, Cronbach’s alpha was calculated to assess the internal consistency of the ItFNAT scores. The overall Cronbach’s alpha for all three versions was 0.882, with individual values of 0.935 for v1, 0.852 for v2, and 0.868 for v3. In Table 3 all the Cronbach’s alpha are reported.

**Table 2:** Principal component analysis for immediate recall (IR), delayed free recall (DFR), and delayed total recall (DTR) scores for each version of the ItFNAT

	Principal component analysis					
	Version 1 *Loadings*	*Uniqueness*	Version 2 *Loadings*	*Uniqueness*	Version 3 *Loadings*	*Uniqueness*
Immediate Recall	0.890	0.207	0.887	0.213	0.876	0.233
Delayed Free Recall	0.955	0.088	0.868	0.247	0.920	0.153
Delayed Total Recall	0.844	0.294	0.792	0.373	0.788	0.380
Overall	Eigenvalue: 2.411 Prop Var: 0.804		Eigenvalue: 2.167 Prop Var: 0.722		Eigenvalue: 2.234 Prop Var: 0.755	

**Table 3:** Cronbach’s alpha for each score and for each version of the ItFNAT

	Cronbach’s alpha			
	Version 1	Version 2	Version 3	Overall
Immediate Recall	0.826	0.641	0.568	0.670
Delayed Free Recall	0.913	0.769	0.789	0.772
Delayed Total Recall	0.705	0.567	0.716	0.707
Overall	0.935	0.852	0.868	0.935

Before proceeding with ItFNAT scores analysis, IR, DFR and TDR normality was assessed using the Shapiro-Wilk Test. The results showed that none of the scores were normally distributed (all *p* <.05). Descriptive statistics and Shapiro-Wilk test results for each ItFNAT score are reported in Table 4.

**Table 4:** Mean, median, standard deviation (SD), interquartile range (IQR) 5th percentile and Shapiro-Wilk test statistics for immediate recall, delayed free recall, and delayed total recall scores (number of correctly recalled face-name associations out of a maximum of 16) of the ItFNAT

	Immediate recall	Delayed free recall	Delayed total recall
*Mean*	6.02	7.26	13.49
*Median*	6.00	7.00	14.00
*SD*	3.32	4.26	2.39
*IQR*	4.00	6.00	2.00
*5th percentile*	2.00	1.00	9.00
*Shapiro-Wils Test*	W = 0.918, *p* <.001	W = 0.956, *p* =.002	W = 0.862, *p* <.001

Accordingly, Kruskal-Wallis Analysis of Variance (ANOVA) was performed for each of the three ItFNAT scores (IR, DFR, and DTR) to assess whether scores varied significantly across test versions (v1, v2, v3). The results indicated no significant differences in the IR score (H(2, *N* = 101) = 2.036, *p* =.361), the DFR score (H(2, *N* = 101) = 1.956, *p* =.376), or the DTR score (H(2, *N* = 101) = 2.223, *p* =.329), suggesting that test versions did not have a significant impact on any of the ItFNAT scores. Furthermore, Levene’s test for equality of variances was performed to assess whether the assumption of equal variances across test versions was held. The results showed no significant differences in the variances for any of the ItFNAT scores (IR: F_2, 98_ = 1.575, *p* =.212; DFR: F_2, 98_ = 2.345, *p* =.101; DTR: F_2, 98_ = 1.366, *p* =.260) between the three versions. All the statistics are reported in Table 5.

**Table 5:** Mean, median, standard deviation (SD), and interquartile range (IQR) and 5th percentile for immediate recall, delayed free recall, and delayed total recall scores (number of correctly recalled face-name associations out of a maximum of 16) across three versions (v1, v2, and v3) of the ItFNAT. Kruskal-Wallis ANOVA and Levene’s test results are also reported for each recall type, assessing differences in recall performance and variance homogeneity across versions

	Immediate Recall			Delayed Free Recall			Delayed Total Recall		
	v1	v2	v3	v1	v2	v3	v1	v2	v3
*Mean*	6.03	6.51	5.59	7.62	7.67	7.15	13.32	13.85	13.38
*Median*	5.00	6.00	6.00	6.00	7.00	6.50	14.00	14.00	14.00
*SD*	4.05	3.11	2.65	4.73	3.80	3.70	2.53	2.08	2.74
*IQR*	4.00	2.00	4.00	6.75	5.00	6.00	3.00	2.00	3.00
*5th percentile*	2.00	2.60	1.65	0.00	4.00	1.65	8.65	9.60	7.95
*Kruskal-Wallis ANOVA*	H = 2.036, *p* =.361			H = 1.956, *p* =.376			H = 2.223, *p* =.329		
*Leven’s test*	F = 1.575, *p* =.212			F = 2.345, *p* =.101			F = 1.366, *p* =.260		

Then, we evaluated the relationship between the sociodemographic variables of our sample and ItFNAT scores. We first conducted a preliminary test for significant differences in age and years of education between males and females using independent samples t-tests. No statistically significant differences were found in age (*p* =.272) or education (*p* =.919) between males and females. However, a significant negative correlation was observed between age and years of education (*r* = −.632, *p* <.001). Consequently, three non-parametric analyses were conducted to assess the influence of participants’ sex, age and education, on each of the ItFNAT scores (see Table 6). Specifically, the Kruskal-Wallis test was used to compare ItFNAT scores between males and females, while the relationship between age, education, and ItFNAT scores was assessed using a 2 × 3 Spearman’s rank correlation matrix. In this latter analysis, the False Discovery Rate was controlled using the Benjamini-Hochberg correction for multiple comparisons. The results showed that sex did not influence any ItFNAT score (*p* >.218). However, significant correlations were found between years of education and each of the ItFNAT scores (all corrected *p* <.022). Furthermore, a significant negative correlation was found between DTR score and age (corrected *p* =.036).

**Table 6:** Results of the Kruskal-Wallis test (H) comparing ItFNAT scores between males and females, and Spearman’s rank correlations (ρ) assessing relationships between age, education and ItFNAT scores. Only significant (< 0.05) Benjamini-Hochberg corrected p-value are reported

	Immediate recall	Delayed free recall	Delayed total recall
Male vs. Female	H (1, *N* = 101) = 1.516	H (1, *N* = 101) = 0.143	H (1, *N* = 101) = 0.872
Age (in years)	ρ = − 0.184	ρ = − 0.174	ρ = − 0.255, corr *p* =.036
Education (in years)	ρ = 0.254, corr *p* =.030	ρ = 0.341, corr *p* <.001	ρ = 0.252, corr *p* =.022

**Fig. 1:**
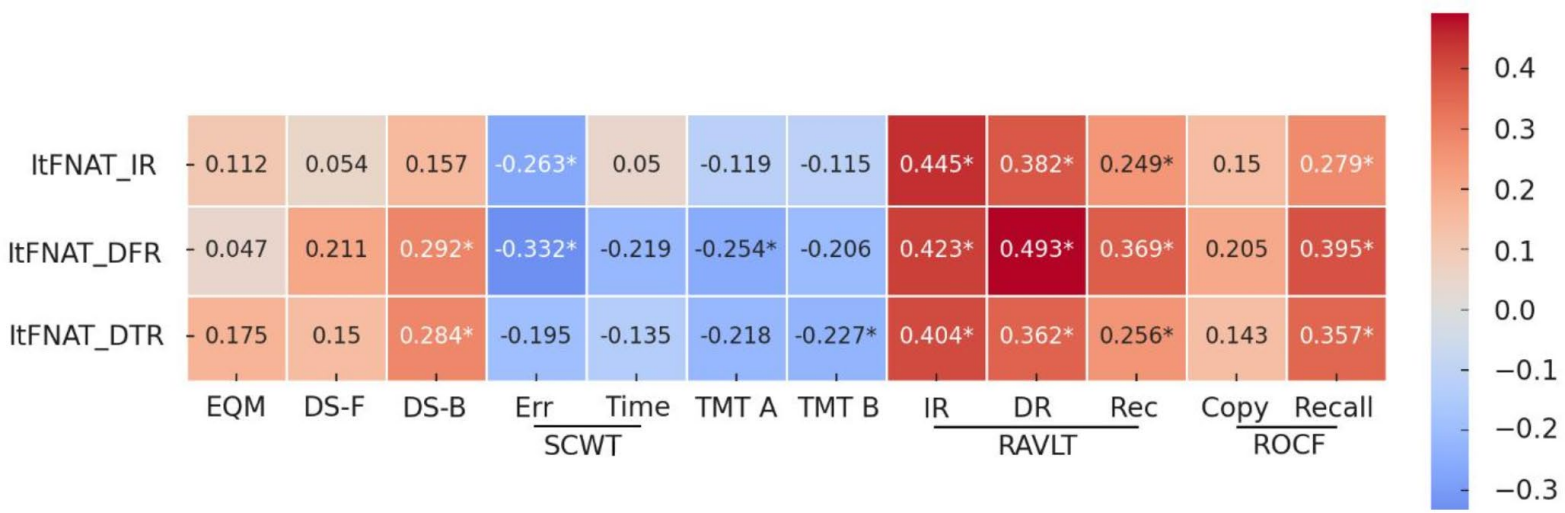
Heatmap of the 3 × 12 Spearman’s rank correlation matrix, displaying Spearman’s rho values for each pairwise. Asterisk (*) indicates a corrected p-value < 0.05

Finally, a 3 × 12 Spearman’s Rank correlation analysis was conducted to examine whether each of the three ItFNAT scores (IR, DFR, DTR) correlated with scores from other cognitive and memory tests (EMQ, DS-F, DS-B, SCWT error, SCWT time, TMT-A, TMT-B, RAVLT immediate recall, delayed recall, recognition, ROCF copy, and recall (see Table 7). To control multiple comparisons, FDR correction with Holm-Bonferroni adjustment was applied to the correlation p-values. The IR score showed significant positive correlations with RAVLT immediate recall (ρ = 0.445, *p* <.001, corrected *p* =.001), delayed recall (ρ = 0.382, *p* <.001, corrected *p* =.001), recognition (ρ = 0.249, *p* =.012, corrected *p* =.025) and ROCF recall (ρ = 0.279, *p* =.005, corrected *p* =.014), all remaining significant after correction. A negative correlation with SCWT error (ρ = − 0.263, *p* =.008, corrected *p* =.021) was also significant after correction. DFR score was positively correlated with DS backward (ρ = 0.292, *p* =.003, corrected *p* =.010), RAVLT immediate recall (ρ = 0.423, *p* <.001, corrected *p* =.001), delayed recall (ρ = 0.493, *p* <.001, corrected *p* =.010), recognition (ρ = 0.369, *p* <.001), and ROCF recall (ρ = 0.395, *p* <.001). Furthermore, significant negative correlations for DFR score were observed with SCWT time (ρ = − 0.321, *p* <.001, corrected *p* =.001), and TMT A (ρ = − 0.254, *p* =.010, corrected *p* =.023). Finally, DTR score showed significant positive correlations with DS backward (ρ = 0.284, *p* =.004, corrected *p* =.012) RAVLT immediate recall (ρ = 0.404, *p* <.001, corrected *p* =.001), delayed recall (ρ = 0.362, *p* <.001, corrected *p* =.001), recognition (ρ = 0.256, *p* =.010, corrected *p* =.023) and ROCF recall (ρ = 0.357, *p* <.001, corrected *p* =.001). Significant negative correlations were observed with TMT B (ρ = − 0.227, *p* =.022, corrected *p* =.044). Other correlations were not significant after adjustment. The heatmap of the correlations are reported in Fig. 1.

**Table 7:** Spearman’s rank correlation coefficients (ρ) between ItFNAT memory scores (Immediate recall, delayed free recall, and delayed total Recall) and cognitive measures, including everyday memory questionnaire (EMQ), digit span (Forward and Backward), Stroop Color-Word Test (SCWT) Errors and Time, Trail Making Test A and B, Ray Auditory Verbal Learning Test (RALT) Immediate recall, delayed recall, and Recognition, and Ray-Osterrieth Complex Figure (ROCF) Copy and Recall. Uncorrected p-values and only significant (< 0.05) Benjamini-Hochberg corrected p-values (Corr p-value) are reported

	Immediate Recall			Delayed Free Recall			Delayed Total Recall		
	*Spearman’s ρ*	*p-value*	*Corr p-value*	*Spearman’s ρ*	*p-value*	*Corr p-value*	*Spearman’s ρ*	*p-value*	*Corr p-value*
EMQ	0.112	0.264	-	0.047	0.638	-	0.175	0.080	-
Digit Span-Forward	0.054	0.592	-	0.211	0.034	-	0.150	0.135	-
Digit Span-Backward	0.157	0.116	-	0.292	0.003	0.001	0.284	0.004	0.012
SCWT_Errors	-263	0.008	0.021	− 0.332	< 0.001	0.010	− 0.195	0.051	-
SCWT_Time	0.050	0.620	-	− 0.219	0.027	-	− 0.135	0.178	-
Trial Making Test A	-119	0.234	-	− 0.254	0.010	0.023	− 0.218	0.029	-
Trial Making Test B	− 0.115	0.251	-	− 0.206	0.039	-	− 0.227	0.022	0.044
RAVLT_IR	0.445	< 0.001	< 0.001	0.423	< 0.001	< 0.001	0.404	< 0.001	< 0.001
RAVLT_DR	0.382	< 0.001	< 0.001	0.493	< 0.001	< 0.001	0.362	< 0.001	< 0.001
RAVLT_Rec	0.249	0.012	0.025	0.369	< 0.001	< 0.001	0.256	0.010	0.023
ROCF_Copy	0.150	0.133	-	0.205	0.040	-	0.143	0.154	-
ROCF_Recall	0.279	0.005	0.014	0.395	< 0.001	< 0.001	0.357	< 0.001	< 0.001

## Discussion

This study presents the first standardized face-name associative test for the Italian sample (ItFNAT), which was designed to assess cross-modal associative memory. The test is freely available (10.6084/m9.figshare.28113620) and consists of three parallel versions. All versions demonstrated high reliability, with good internal consistency and no significant score differences, underscoring the test’s robustness and suitability as a standardized tool within the Italian context. The scores of the test, namely Immediate Recall (IR), Delayed Free Recall (DFR), and Delayed Total Recall (DTR), offer distinct insights into cognitive functioning. While all the scores reflect both verbal and visual memory performance and reflect a shared underlying cognitive construct, DFR and DTR also capture broader aspects of general cognitive abilities, such as attention and working memory, respectively. This pre-validation process allowed for the derivation of preliminary descriptive statistics. However, the recruitment of a larger sample is necessary, given that sociodemographic factors such as age, and particularly years of education, were found to influence ItFNAT scores.

The internal consistency of the ItFNAT was assessed using Cronbach’s alpha, demonstrating good overall reliability (0.88). Version-specific alphas were all strong (> 0.85). Regarding the scores, the IR score had slightly lower alphas (0.57–0.83), while the DFR and DTR scores demonstrated higher consistency (> 0.77). Additionally, principal component analysis indicated that the three scores of each version loaded (> 0.81) onto a single component, explaining about 76% of each version variance. These findings suggest that the ItFNAT is a reliable measure of face-name associative memory, with strong internal consistency across each score and version. Overall descriptive statistics revealed that IR score averaging around 6 out of 16, DFR around 7, and DTR score around 13. Despite several studies suggested that minimal forgetting can occur after 30 min from encoding [21], this phenomenon was not observed in the present study, where the DFR score median and mean was slightly higher than the IR score. This phenomenon can be attributed to the shorter interval (15 min) between the immediate and delayed recall trials. Additionally, during the immediate recall trial, the correct face-name association was re-presented to participants regardless of their IR accuracy response, thereby reinforcing the name-face association. The use of a recognition trial when participants were unable to freely recall a name during the delayed recall trials significantly enhanced performance, increasing the number of correctly recalled associations. This finding is consistent with the efficacy of associative prompts, which leverage recognition processes, in enhancing free memory recall among the general population [35]. Thanks to the recognition trial, the ItFNAT can potentially distinguish retrieval versus encoding deficits, aiding differential diagnosis of AD and other conditions. In AD, limited cue benefits suggest encoding issues [13], whereas larger improvements in conditions like frontotemporal dementia [25] or neurodevelopmental disorders (e.g., Attention-Deficit/Hyperactivity Disorder [31] and indicate retrieval deficits. Cue-based strategies could further enhance cognitive rehabilitation in early AD [11] and help monitor progression in neurodegenerative disorders. The inclusion of the 5th percentile value within our descriptive statistics provided a preliminary cutoff (2 for IR, 1 for DFR, and 9 for DTR) indicating potential cognitive concerns. These cutoff points will help guide future research and clinical assessments.

Regarding the comparability of the test versions (v1, v2, v3), the results indicated no significant differences in the scores across versions, suggesting that the different versions of the test produced comparable results. This finding was confirmed by the Kruskal-Wallis ANOVA, which showed no significant variations in the IR, DFR, or DTR scores between the three versions and by Levene’s test for equality of variances further supporting the consistency of the test versions. These results ensure that any differences in scores observed across participants are likely attributable to individual differences rather than the test version itself. Within the individual factors influencing ItFNAT scores, the participants’ educational attainment demonstrates to be the most important, while age plays a role mainly for TDR scores. Participants with higher levels of education reported higher IR, DFR and DTR scores. No significant effects were observed for sex on any of the ItFNAT scores. These findings align with the existing literature, which suggests that individuals with higher educational attainment may possess enhanced associative memory capabilities or employ more effective encoding strategies. This is also consistent with findings of previous research linking higher education level to improved performance on cognitive tasks [19] and its neuroprotective role [6]. The negative correlations between participants’ age and DTR can be at least partially account to the global increase in educational attainment [33], However, it is well known that even in healthy aging, a decline in cognitive functioning is typically observed across different cognitive domains [29] and that this decline can be modulated by education [1]. To ensure more precise assessments, future studies should stratify the sample by age and education to provide more accurate reference values.

Principal component analysis revealed that the three scores all loaded onto a single component, although DTR exhibited a moderate degree of uniqueness. This was consistent with the 3 × 12 Spearman’s correlation matrix, which showed that IR, DFR, and DTR scores positively correlated with both verbal and visual memory tests, while each score also displayed distinct relationships with attention and working memory indices. Specifically, the ItFNAT IR score demonstrated positive correlations with RAVLT Immediate and Delayed Recall, with RAVLT Recognition, as well as ROCF Recall. Additionally, it showed a negative correlation with the number of errors on the SCWT, suggesting that cognitive control abilities play a pivotal role in ItFNAT IR performance. The ItFNAT DFR score exhibited the same correlations as the IR score, along with a positive correlation with DS-B score and a negative correlation with the time to complete the TMT-A. This suggests that faster visuo-spatial speed of processing and stronger phonological working memory are associated with better DFR performance. This finding highlights the role of DFR as a robust indicator of general cognitive functioning, integrating attentional processes, working memory, and cross-modal memory. Finally, the DTR score demonstrated negative correlations with the time required to complete TMT-B and positive correlations with DS-B, ROCF Recall, and all RAVLT scores. This highlights its ability to assess not only working and associative memory processes but also visuo-spatial attentional capacities. Conversely, any ItFNAT score exhibited correlations with ROCF Copy, DS-F, EMQ scores, or SCWT Time. Overall, these results underscore the utility of the ItFNAT as a comprehensive tool for assessing cross-modal associative memory and its ability to capture other cognitive domains such as attention processes and working memory [24]. Actually, both attentional process and working memory plays a critical role in facilitating efficient encoding and retrieval of information [16, 23], particularly in older adults [10, 28].

### Limitations, future research and conclusions

Notwithstanding the encouraging results, this preliminary validation study is not without limitations. For instance, the IR score of Version 3 and the DTR score of Version 2 exhibited low internal consistency. This may be partially attributed to the dichotomous scoring method (correct vs. incorrect) employed, the low number of items (16) and the relatively small sample size of approximately 33 individuals per version. A larger sample size could help refine these measures. Additionally, the assessment of test-retest reliability is crucial to evaluate the consistency of the tool over time. Furthermore, while the sample size was adequate for preliminary validation, it did not fully capture the variability in memory performance across a broader range of demographics, particularly among older adults and individuals with lower educational attainment. Future research could enhance the clinical applicability of the ItFNAT by expanding normative data to include a wider range of ages and educational backgrounds, thereby refining cut-off scores for different groups or providing correction grids.

Finally, while the present study focused exclusively on correct responses, it is possible that participants may choose not to respond (i.e., “missed”) or provide incorrect answers. These latter incorrect answers can be intrusive (associating to a face a name never encountered during the former phases) or interfering (associating a face with a name that was incorrect but presented during the former phases). The analysis of these error types could yield valuable insights, which could assist in distinguishing between different diagnostic categories in the context of neurodegenerative or neurodevelopmental conditions. Future studies could explore whether specific response patterns are linked to distinct conditions, offering a deeper understanding of underlying cognitive processes and their relationship to different clinical profiles.

In conclusion, this study provides a standardized version of a FNAT validated on an Italian population. The ItFNAT is a rapid instrument designed to evaluate cognitive functioning with a particular emphasis on cross-modal associative memory. A significant strength of the ItFNAT is its availability in three parallel versions, all of which are freely accessible online. These versions have been developed to minimize potential learning effects when employed repeatedly for the purpose of monitoring cognitive changes over time. This feature is particularly advantageous in clinical trials, where repeated assessments are common practice. As neurodegenerative diseases, including Alzheimer’s disease, continue to rise on a global scale, standardized tools such as the ItFNAT will play a pivotal role in the early detection of cognitive changes [5, 26], guiding both clinical diagnosis and research into therapeutic interventions.

## Data Availability

The data that support the findings of this study and the ItFNAT are freely available here: 10.6084/m9.figshare.28113620.
